# Data on morphotectonic indices of Dashtekhak district, Iran

**DOI:** 10.1016/j.dib.2017.08.052

**Published:** 2017-09-05

**Authors:** Ali Fadaie Kermani, Reza Derakhshani, Shahram Shafiei Bafti

**Affiliations:** Department of Geology, Shahid Bahonar University of Kerman Iran

**Keywords:** Tectonics, Geomorphology, Morphometrics, Geology, Kerman

## Abstract

Morphotectonic indices by representing the longer period of time than recorded earthquake data, are useful in evaluating the tectonic activity of a region. Dashtkhak area is located in Kerman province of Iran, where one of the most active faults, Kouhbanan strike slip fault, passes through. This data article provides a precise level data on mountain fronts and valleys of Dashtkhak region that is fundamental for morphotectonic investigations of the relationship among geomorphology and tectonic activity. This data is valuable in the field of geology and geography. Mountain fronts and valleys data is more relevant in the field of tectonics and geomorphology. It helps to evaluate a region from the viewpoint of tectonic activity. The data which are presented for 31 mountain fronts and 61 valleys, is taken by processing of remotely sensed Landsat satellite data, photogeology of areal photographs, measuring on topographic maps and controlled by field checking. This data is useful for calculating of some morphotectonic indices such as sinuosity of mountain fronts (*s*_mf_), mountain front faceting percentage (Facet%), the ratio of valley floor width to valley height (*V*_f_) and the valley ratio (*V*).

**Specifications Table**

**Table:** 

Subject area	*Geology*
More specific subject area	*Tectonics, Morphotectonics, Tectonic geomorphology*
Type of data	*Table*
How data was acquired	*Survey, Topographic maps, Photogeology, Field checking*
Data format	*Raw, analyzed*
Experimental factors	*Geometry of Mountain fronts and valleys*
Experimental features	*Mountain fronts and valleys*
Data source location	*Dashtekhak, Zarand, Iran.Latitude: 56°,19*′ *to 56°,48*′ *N & Longitude: 30°,46′ to 31°,14′ E*
Data accessibility	*Data is available with this article.*

**Value of the data**

• The data provides a vivid vision about activity stage of Dashtekhak area.
• It helps to explain the impact of Kouhbanan fault segments on the activity of the area.
• Data can be utilized for quantitative analysis in the field of tectonic geomorphology and morphotectonics.
• Other researchers may utilize the data for their research work and further investigation.

## 1 Data

The data presented here describes the morphometric characteristic of 31 mountain fronts and 61 valleys of Dashtekhak district. Data is given in table form.The data is prepared on the basis of field work and laboratory analysis.

## 2 Experimental design, materials and methods

Morphotectonic investigation is an effective tool to enable us to detect and distinguish the procedures that occur on the landforms. Erosion and tectonic movements leave their imprints as a morphological components which their measurement is the best approach to relate the landforms with the neotectonic evolution of the area [1], [2].

In order to achieve the most accurate data, the analysis was undertaken on topographic maps in 1:10,000 scale. Landsat Satellite images and 1:50,000 scale aerial photographs beside advantages of google earth software are used to locate and measure precisely the mountain fronts and valleys geomorphic specifications. The analysis comprised the calculation of morphotectonic indices (*s*_mf_, Facet%, *V*_f_, *V*) for 31 mountain fronts and 61 valleys, according to the mathematical relationships which are presented on Table 1, Table 2, Table 3, Table 4, Table 5.

**Table 1:** Formulas for calculating the morphotectonic indices. *S*_mf_: sinuosity of mountain fronts, *L*_mf_: mountain front length along the foot of the mountain, *L*_s_: the length of the straight line of the mountain front, Facet%: the percentage of the mountain front faceting, *L*_f_: total length of facets in a mountain front, *V*_f_: the valley floor width to height ratio, *V*_fw_: the width of the valley floor, *E*_ld_: the elevation of the left valley divides, *E*_rd_: the elevation of the right valley divides, *E*_sc_: the elevation of the valley floor, *V*: Valley ratio, *A*_v_: the area of the valley, *A*_c_: the area of the semi-circle with an equivalent radius of valley depth.

Morphotectonic index	Formula
*s*_mf_	*S*_mf_=*L*_mf_/*L*_s_
Facet%	Facet%=(*L*_f_/*L*_s_)
*V*_f_	*V*_f_=2*V*_fw_/[(*E*_ld_-*E*_sc_)+(*E*_rd_−*E*_sc_)]
*V*	*V*=*A*_v_/*A*_c_

**Table 2:** Data which is used for determination of mountain front sinuosity index.

**Front no.**	***L***_**mf**_**(m)**	***L***_**s**_**(m)**	***S***_**mf**_
F1	482	381	1.27
F2	164	148	1.11
F3	845	589	1.43
F4	912	673	1.36
F5	441	323	1.37
F6	1092	914	1.19
F7	837	684	1.22
F8	2078	1628	1.28
F9	1084	906	1.20
F10	1100	941	1.17
F11	2029	1818	1.12
F12	1693	1315	1.29
F13	1696	1514	1.12
F14	1742	1439	1.21
F15	3789	2919	1.30
F16	601	517	1.16
F17	1580	1377	1.15
F18	1627	1181	1.38
F19	1471	1310	1.12
F20	885	692	1.28
F21	2265	1772	1.28
F22	778	597	1.30
F23	2663	2002	1.33
F24	1872	1668	1.12
F25	2616	2258	1.16
F26	7308	6332	1.15
F27	2389	1980	1.21
F28	2894	2343	1.24
F29	807	712	1.13
F30	936	870	1.08
F31	726	832	0.87

**Table 3:** Data which is used for determination of mountain front faceting index.

Front no.	*L*_s_ (m)	*L*_f_ (m)	Facet%
F1	381	325.4	85.4%
F2	148	137	92.6%
F3	589	504.4	85.6%
F4	673	637.5	94.7%
F5	323	263.9	81.7%
F6	914	832	91.0%
F7	684	568	83.0%
F8	1628	1452	89.2%
F9	906	837	92.4%
F10	941	936	99.5%
F11	1818	1678	92.3%
F12	1315	1278	97.2%
F13	1514	1305	86.2%
F14	1439	1367	95.0%
F15	2919	2696	92.4%
F16	517	516	99.8%
F17	1377	1332	96.7%
F18	1181	1016	86.0%
F19	1310	1297	99.0%
F20	692	631	91.2%
F21	1772	1656.8	93.5%
F22	597	551	92.3%
F23	2002	1968.2	98.3%
F24	1668	1524	91.4%
F25	2258	2068	91.6%
F26	6332	5939	93.8%
F27	1980	1930	97.5%
F28	2343	2089	89.2%
F29	712	646	90.7%
F30	870	542	62.3%
F31	832	511	61.4%

**Table 4:** Data which is used for determination of *V*_f_ index.

Valley no.	*E*_ld_	*E*_rd_	*E*_sc_	*V*_fm_	*V*_f_
1	2094	2112	2031	61	0.85
2	2210	2269	2122	101	0.86
3	2438	2338	2243	74	0.51
4	2092	2044	2021	41	0.87
5	2228	2225	2134	76	0.82
6	2335	2395	2250	158	1.37
7	2459	2524	2373	131	1.11
8	2155	2138	2089	39	0.68
9	2283	2226	2178	50	0.65
10	2460	2417	2320	123	1.04
11	2261	2241	2221	26	0.87
12	2273	2275	2167	111	1.04
13	2439	2439	2334	67	0.64
14	2400	2404	2348	48	0.89
15	2365	2382	2245	73	0.57
16	2343	2367	2224	114	0.87
17	2270	2283	2212	75	1.16
18	2284	2294	2181	95	0.88
19	2452	2418	2346	82	0.92
20	2485	2541	2418	133	1.40
21	2475	2472	2291	104	0.57
22	2093	2116	2029	80	1.06
23	2150	2223	2050	98	0.72
24	2300	2286	2248	162	3.60
25	2225	2229	2168	67	1.14
26	2241	2199	2160	83	1.38
27	2318	2301	2261	71	1.46
28	2173	2166	2119	55	1.09
29	2124	2142	2070	76	1.21
30	2161	2184	2103	137	1.97
31	2131	2140	2097	71	1.84
32	2118	2076	2039	65	1.12
33	2076	2115	2048	101	2.13
34	2266	2239	2091	170	1.05
35	2061	2070	2002	88	1.39
36	2102	2095	2047	89	1.73
37	2211	2244	2153	113	1.52
38	2070	2074	2051	54	2.57
39	2170	2139	2119	106	2.99
40	2286	2283	2254	67	2.20
41	2104	2088	2053	113	2.63
42	2152	2177	2110	148	2.72
43	2196	2201	2131	122	1.81
44	2182	2188	2150	96	2.74
45	2164	2186	2137	56	1.47
46	2095	2096	2070	93	3.65
47	2245	2224	2164	138	1.96
48	2186	2114	1985	186	1.13
49	2240	2218	2151	65	0.83
50	2346	2321	2253	107	1.33
51	2221	2213	2099	329	2.79
52	2258	2294	2173	99	0.96
53	2385	2351	2265	132	1.28
54	2470	2449	2386	107	1.46
55	2349	2344	2289	81	1.41
56	2189	2205	2106	260	2.86
57	2256	2256	2160	168	1.75
58	2220	2215	2145	127	1.75
59	2397	2326	2219	96	0.67
60	2477	2591	2249	126	0.44
61	2339	2331	2237	50	0.51

**Table 5:** Data which is used for determination of *V* ratio index.

Valley no.	*A*_v_	h	*A*_c_	*V* ratio
1	3636	61	5842	0.62
2	6480	87	11,883	0.55
3	8284	90	12,717	0.65
4	278	16	402	0.69
5	6923	80	10,048	0.69
6	10,847	85	11,343	0.96
7	8101	88	12,158	0.67
8	1498	49	3770	0.40
9	2265	48	3617	0.63
10	7482	97	14,772	0.51
11	211	15	353	0.60
12	8179	105	17,309	0.47
13	7392	106	17,641	0.42
14	2187	52	4245	0.52
15	14,598	120	22,608	0.65
16	14,755	119	22,233	0.66
17	3971	58	5281	0.75
18	11,190	103	16,656	0.67
19	4715	72	8139	0.58
20	6640	67	7048	0.94
21	26,013	181	51,435	0.51
22	4620	64	6431	0.72
23	9863	100	15,700	0.63
24	2309	38	2267	1.02
25	4205	57	5101	0.82
26	1776	39	2388	0.74
27	2028	40	2512	0.81
28	2416	47	3468	0.70
29	3863	54	4578	0.84
30	3902	58	5281	0.74
31	1377	34	1815	0.76
32	1657	37	2149	0.77
33	1065	28	1231	0.87
34	28,612	148	34,389	0.83
35	5284	59	5465	0.97
36	2854	48	3617	0.79
37	4012	58	5281	0.76
38	507	19	567	0.89
39	651	20	628	1.04
40	1139	29	1320	0.86
41	2055	35	1923	1.07
42	2123	42	2769	0.77
43	5109	65	6633	0.77
44	1710	32	1608	1.06
45	1243	27	1145	1.09
46	1068	25	981	1.09
47	4830	60	5652	0.85
48	27,017	129	26,126	1.03
49	5799	67	7048	0.82
50	4624	68	7260	0.64
51	19,535	114	20,404	0.96
52	9052	85	11,343	0.80
53	9107	86	11,612	0.78
54	13,919	103	16,656	0.84
55	3525	55	4749	0.74
56	11,173	83	10,816	1.03
57	15,774	96	14,469	1.09
58	5679	70	7693	0.74
59	10,109	107	17,975	0.56
60	41,221	228	81,615	0.51
61	7950	94	13873	0.57

The sinuosity and faceting of mountain fronts are useful morphotectonic indices that reflect the balance between erosional and tectonic forces. So that, if the tectonic activity decreases, erosion process begins to form the sinusoidal mountain fronts which turns more irregular over time. For S_mf_ the closer the data to 1.0 is thought to be a tectonically highly active mountain front while the higher values belong to less active regions. For Facet% index, large percentages reflect high level of tectonic activity in the mountain fronts. Therefore, the mountain fronts influenced by active uplifting are almost straight and their *S*_mf_ indices roughly equal 1 and Facet% around 100%. *V*_f_ and *V* are valuable morphometric indices to quantitatively differentiate the shape of a valley along its cross section. High values of these indices indicate U-shaped valleys that associated with low tectonic activity, while low values show deep V-shaped ones indicating active uplift [3], [4], [5], [6], [7], [8], [9].

## Funding sources

This work is part of a MSc thesis of Ali Fadaie Kermani and was funded by Shahid Bahonar University of Kerman, Iran (93127003).

## Figures and Tables

**Fig S1:**
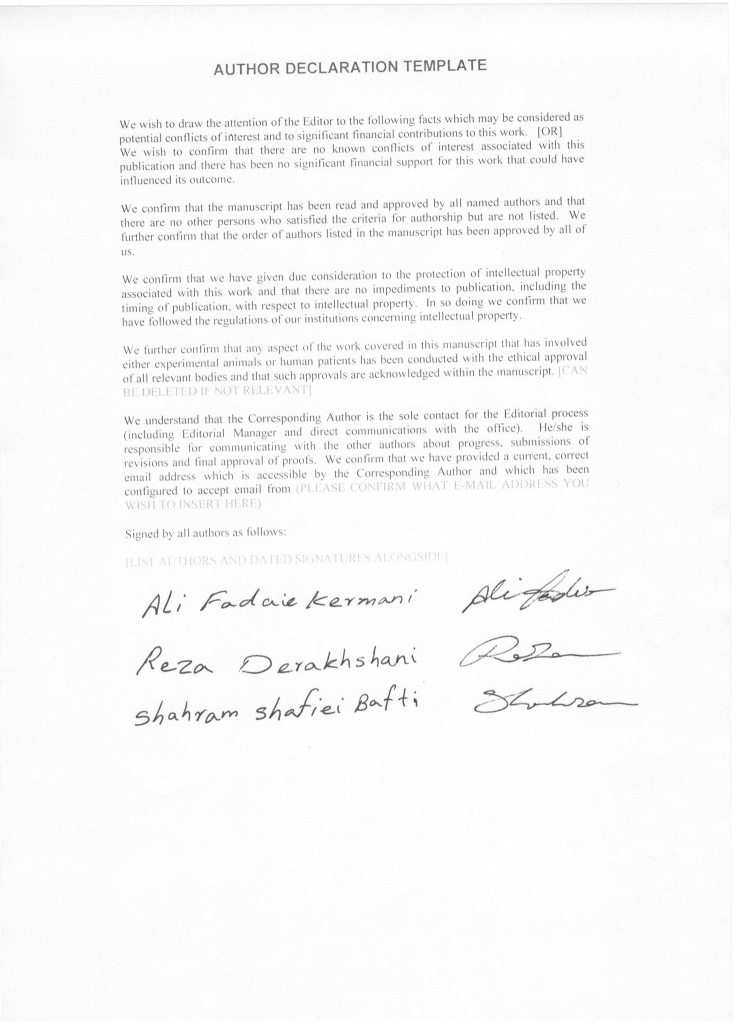
.
